# Pathways and Genetic Determinants of Impulse Control Disorders in Parkinson’s Disease

**DOI:** 10.3390/life16060897

**Published:** 2026-05-27

**Authors:** Kallirhoe Kalinderi, Vasileios Papaliagkas, Oraiozili Goula, Liana Fidani, Maria Chatzidimitriou

**Affiliations:** 1 Department of Biomedical Sciences, Faculty of Health Sciences, International Hellenic University, 57400 Thessaloniki, Greece; vpapaliagkas@ihu.gr (V.P.); chdimitr@ihu.gr (M.C.); 2 School of Medicine, Faculty of Health Sciences, Aristotle University of Thessaloniki, 57400 Thessaloniki, Greece; goularoz@gmail.com; 3 Laboratory of Medical Biology-Genetics, School of Medicine, Faculty of Health Sciences, Aristotle University of Thessaloniki, 57400 Thessaloniki, Greece; sfidani@auth.gr

**Keywords:** Parkinson’s disease, gene, dopamine, pathway, polymorphism, receptor, pathogenesis, serotonin, opioid, glutamine

## Abstract

**Background:** Impulse control disorders (ICDs) are a common non-motor complication in Parkinson’s disease (PD) patients with multiple negative consequences for the individual and caregivers. Although ICDs are strongly linked to dopaminergic therapy, particularly dopamine agonists, only a percentage of patients develop these behaviors, suggesting the involvement of additional susceptibility factors, including genetic variability. This review aims to analyze current knowledge on the genetic background of ICDs. **Methods:** A literature search was conducted in the PubMed and Scopus databases for peer-reviewed research regarding the role of genetics in ICDs, published in the English language from 1996 to 2026. References of the selected articles for possible additional articles were also screened in order to include most of the key recent evidence. Genes that are involved in the dopaminergic system play a central role in ICD susceptibility, although the findings in studies are often inconsistent and not replicated. Moreover, variants in genes related to the glutamatergic (e.g., *GRIN2B*), serotonergic (e.g., *HTR2A* and *TPH2*), and opioid systems (e.g., *OPRK1* and *OPRM1*) have been implicated, supporting a multi-system contribution to ICD pathophysiology. **Conclusions:** Early recognition of genetic factors that increase susceptibility to ICDs in PD patients is awaited to increase diagnostic accuracy and expedite individualized treatment.

## 1. Introduction

Parkinson’s disease (PD) is the second most common neurodegenerative disorder with a prevalence of about 1% in people over 60 years of age and about 4% in people over 85 years old [1]. It is characterized by the progressive loss of dopaminergic neurons in the substantia nigra pars compacta and the presence of Lewy bodies (LBs) in the surviving neurons. The motor symptomatology of the disease, including resting tremor, bradykinesia, rigidity, postural instability, and its responsiveness to L-dopa therapy, is well known. Although the motor manifestations of PD are well established, non-motor symptoms are increasingly recognized as major contributors to disease burden and often precede motor onset [2,3,4].

Among these, impulse control disorders (ICDs) represent a particularly disabling group of behavioral complications. ICDs are characterized as an inability to resist the impulse, drive or temptation to engage in repetitive, excessive and compulsive behaviors, despite their possible negative effects. ICDs include, among others, compulsive eating, shopping or medication use, pathologic gambling, hypersexuality and punding [5]. The prevalence of ICDs in PD patients demonstrates a wide variability from 3.5 to 42.8%, reflecting substantial heterogeneity across studies that can be attributed to different study designs, assessment tools (e.g., QUIP vs. clinical interview), inclusion of broader impulsive–compulsive behaviors or demographic and clinical characteristics [5,6,7]. Notably, PD patients with ICDs suffer more frequently from neuropsychiatric comorbidities, such as depression or anxiety, and PD patients with multiple ICDs have been found to experience more severe dyskinesia problems [8]. Interestingly, ICDs are frequently observed in PD patients treated with dopaminergic agents, especially dopamine agonists [5]; however, not all patients develop these behavioral disorders, suggesting that clinical, genetic or environmental factors may also have a crucial role in the pathogenesis of ICDs. Early recognition of these factors could improve diagnostic accuracy and lead to individualized therapeutic interventions. Emerging evidence indicates that specific variants in genes implicated in different pathways such as dopaminergic, glutaminergic, serotonergic, etc., may play a pivotal role in the genetic susceptibility of an individual to develop ICDs.

The existing studies are often limited by small sample sizes, lack of replication across diverse populations, and heterogeneous definitions of ICD phenotypes, resulting in contradictory findings. Moreover, most of the available review studies focus on the descriptive reporting of individual genetic associations, without correlation with the pathophysiological pathways that are involved. As a result, the translation of these genetic findings to clinical practice remains limited.

Addressing these gaps is of clinical importance, as better knowledge of genetic susceptibility might aid in the early identification of PD patients at higher risk of developing ICDs, especially in terms of dopaminergic therapy. This knowledge could improve risk stratification and guide treatment selection (e.g., careful use of dopamine agonists), as well as enable closer behavioral monitoring, ultimately supporting a precision medicine approach.

The present review aims to provide a structured and critically appraised synthesis of the genetic evidence underlying ICDs in PD. In contrast to previous narrative reviews, we emphasize the strengths and limitations of existing studies and propose a pathophysiological framework with potential clinical implications that links multiple neurotransmitter pathways involved in impulse regulation (Table 1).

## 2. Methods

This study was conducted as a structured narrative review, incorporating methodological elements adapted from the PRISMA guidelines to enhance transparency and reproducibility. In March 2026, a comprehensive literature search was conducted to identify original research studies (e.g., case–control, GWAS, and cohort studies) within the PubMed and Scopus databases from January 1996 until January 2026. Boolean operators (AND and OR) were used to search by a combination of these keywords: “Parkinson’s disease”, “impulse control disorders”, “gene”, “genetic polymorphism”, “SNP”, “genetic variant”, and “genetics”. Studies were included if they: (a) investigated genetic factors associated with ICDs in PD, (b) included human participants and (c) were written in English. Studies not specifically addressing PD-related ICDs, reviews, editorials, case reports and animal studies were excluded. To ensure the inclusion of high-quality studies, we restricted the selection to those published in peer-reviewed journals. Titles and abstracts were screened independently by two reviewers, followed by full-text assessment of potentially relevant articles. Discrepancies were resolved by discussion between the two reviewers and eventual consensus. When a consensus could not be reached, a third senior reviewer was consulted. Reference lists of the selected studies were also screened to identify additional relevant publications. Duplicate records were removed prior to screening.

Because the selected studies showed great heterogeneity, including differences in study design, sample size, and genetic methodologies, a quantitative risk-of-bias assessment was not applicable. The included evidence was critically based on several parameters, such as sample size, statistically significant associations, replication status and consistency across studies.

## 3. Results and Discussion

Before the presentation of individual genetic associations, it is important to note that the current studies are characterized by substantial heterogeneity. Many studies are based on relatively small cohorts, and the findings are often not replicated across independent populations. In addition, differences in study design, ethnicity, diagnostic criteria, and dopaminergic treatment exposure may contribute to inconsistent results. Therefore, the reported associations should be interpreted with caution, and emphasis should be given on the overall consistency of findings rather than isolated reports.

### 3.1. Database Search

**Figure 1:**
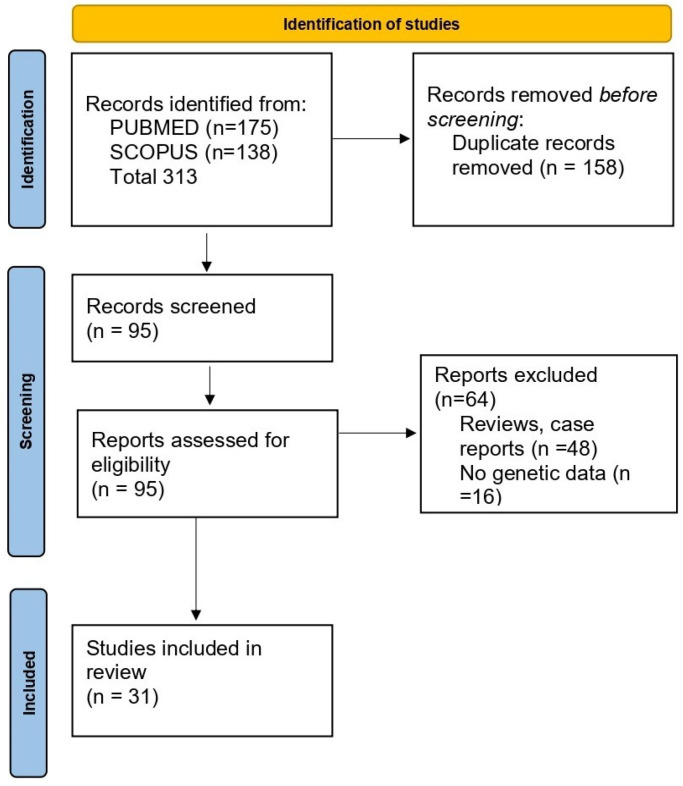
Flowchart of the selection of studies included in the review.

A total of 313 potentially relevant records were retrieved from the PUBMED and SCOPUS databases. After duplicate removal and title/abstract screening, 158 records were excluded, and another 64 records were removed because they were review studies or case reports or they did not include any genetic data. A total of 31 studies were enlisted for the final collection of data. The search flowchart is presented in Figure 1.

### 3.2. Genes Involved in the Dopaminergic Pathway

Recent data indicate that the dopaminergic pathway is not only involved in PD pathogenesis but also in ICDs. Such behavioral addictions are frequently associated with dopamine agonist therapy of PD patients overstimulating the reward-seeking mesolimbic pathway [28]. Several genes that encode for enzymes, transporters, and receptors that participate in dopamine synthesis, transport, degradation and signaling have been found to be associated with the development of ICDs in PD patients [11].

#### 3.2.1. DRD1

Dopamine is synthesized in the dopaminergic neurons from the amino acid Tyrosine in several steps. Levodopa produces dopamine via dopa decarboxylase (DDC). Dopamine binds to dopamine receptors on postsynaptic neurons or glial cells. There are five types of dopamine receptors (DRs): DR1 and DR5 which act as D1-like receptors, and DR2, DR3 and DR4 which act as D2-like receptors. D2-like receptors have a 10- to 100-fold greater affinity for dopamine compared to the D1-like receptors. Moreover, DR1 and DR2 are the most abundant DR subtypes [29].

The dopamine receptor D type 1 (*DRD1*) gene is located on chromosome 5q35.1, has two exons and the encoded protein has 446 amino acids. The DR1 receptor is highly expressed in the brain, especially in the caudate-putamen, the nucleus accumbens, the substantia nigra pars reticulata, and the cerebral cortex; however, it has lower expression in peripheral tissues [29].

Τhe *DRD1* rs4532 and rs4867798 polymorphisms have been associated with ICDs in PD patients. These polymorphisms are located outside of the *DRD1* coding region. Interestingly, the rs4532 polymorphism in the 5′ untranslated region (5′-UTR) has previously been associated with compulsive addictive behavior [9]. In the study by Abidin et al. [10] the T allele was significantly associated with an increased risk of ICDs in PD patients *DRD1* rs4532 T allele (OR  =  21.33; 95% CI, 1.97–230.64; *p*  =  0.0024). However, it should be noted that the study sample was small (52 PD patients), the confidence intervals were wide and similar effect sizes failed to replicate in other studies. Regarding the rs4867798 variant, the C allele also increased the risk of ICDs in PD patients. However, the two variants showed no LD in the subsequent haplotype-based analysis. The two variants in the 5′-UTR and 3′- UTR of *DRD1* may affect mRNA stability, the binding site of microRNA (miRNA) or the secondary structure of mRNA, and thus may have an impact on *DRD1* expression [10].

Another polymorphism in the 5′-UTR of *DRD1*, rs5326, has been associated with an increased risk of ICDs in PD patients (OR 2.9; 95% CI 1.1–7.6; *p* = 0.026) [11]. This variant has also been linked to reduced *DRD1* expression and impaired cognitive function [30,31,32]; however, given the limited evidence, its contribution to ICD susceptibility in PD remains to be confirmed in larger studies.

#### 3.2.2. DRD2/ANKK1

The dopamine receptor D type 2 (*DRD2*) gene is located on chromosome 11q22-23, and is organized in eight exons separated by seven introns and encodes for DRD2 which is highly expressed in the basal ganglia, including the caudate-putamen, the substantia nigra pars compacta and the cerebral cortex. DRD2 is a transmembrane G protein-linked receptor that regulates intracellular signaling by inhibiting cAMP synthesis; it is involved in the mesocorticolimbic pathway and affects motor control [29].

The *DRD2/ANKK1* rs1800497 polymorphism, located close to the coding region of the ankyrin repeat and kinase domain containing 1 gene (*ANKK1*) adjacent to *DRD2*, has been extensively studied. This polymorphism, also referred as Taq1A, causes an amino acid change (Glu713Lys) in ANKK1 [12] and occurs in two alleles (A1 and A2); thus, individuals can have 3 possible genotypes: A1/A1, A1/A2 and A2/A2. The Taq1 A (A1) allele has been associated with lower D2 receptor striatal density and reduced D2 binding in the striatum. More specifically, it has been observed that the D2 receptor density can be reduced by up to 30% in A1 carriers, especially in the ventral regions of the caudate and putamen [33,34]. Additionally, this variant has been implicated in conditions like pathological gambling [35], greater ventral striatal reactivity [36], dependence or polysubstance abuse [37]. Abidin et al., observed that the *DRD2/ANKK1* rs1800497 variant was associated with increased risk of developing PD-ICDs (OR = 3.77; 95% CI, 1.38–10.30; *p* = 0.0044) [10], which is consistent with a previous study in PD-ICD individuals [38]; however, other studies have not replicated these findings [19,39]. These findings should be interpreted with caution, as the same methodological limitations previously discussed for this study also apply here. Interestingly, in another study the rs1800497 Taq1A (A1) polymorphism (A1/A1 or A1/A21) was found to display a better ability to suppress impulsive actions when on dopamine agonist medication. The rs1800497 polymorphism produces a Glu713-to-Lys (E713K) substitution and this significant protein structure modification, from an amino acid group with a negatively charged side chain to one with a positively charged residue, may affect the DRD2 expression and increase the risk of neuropsychiatric symptoms in PD patients [40].

Additionally, in the study by Fedosova et al., which examined 386 PD patients, the TT genotype of the rs6275 substitution in exon 7 of *DRD2* was found to increase ICD risk (*p* = 2.9 × 10^−8^, OR = 9.00, CI95% [3.97–20.14]) [13]. The recessive T allele influences the stability and translation of the protein, and the TT genotype may lead to overactivation of downstream dopamine receptors and an increased response to the released dopamine [41]. Also, the recessive A allele of the rs12364283 substitution was found to be associated with ICD development, as well as the dominant A allele of the rs1076560 substitution in the Intron 6 of *DRD2*, suggesting a possible role of *DRD2* in ICD development [13]. Further studies examining the role of the *DRD2/ANKK1* polymorphisms regarding the risk of ICDs in PD patients should be performed for more definite conclusions to be drawn.

#### 3.2.3. DRD3

The dopamine receptor D type 3 (*DRD3*) gene is located on chromosome 3q13.3. DRD3 is predominantly found in the ventral striatum, the nucleus accumbens, where, with the aid of the dopamine transporter, it regulates dopamine release and clearance. DR3 is also closely associated with the limbic system [29].

The *DRD3* rs6280 polymorphism, a thymine (T) to cytosine (C) change that results in a Ser9-to-Gly substitution, has been examined regarding ICDs. The homozygous glycine variant has greater receptor binding affinity compared to the wild-type homozygous serine variant. Behavioral addictions in PD have been found to be associated with an early onset of PD, the rs6280 *DRD3* variant and the type of dopamine agonist used [42]. The role of this *DRD3* polymorphism has been highlighted in different ethnicities [19,43], however, with variable results, due to ethnic variances or differences in the age at PD onset of the enrolled patients. Moreover, the rs6280 *DRD3* variant has been implicated in aberrant decision-making under uncertainty in 78 PD patients without active ICDs, suggesting that it could affect impulsivity [14]. Apathy and the rs6280 *DRD3* polymorphism have also been found as interactive risk factors for ICD severity. Apathy has been associated with atrophy of the bilateral putamen and reduced dopamine synthesis in the limbic striatum, and PD patients with the *DRD3* risk variant had also reduced dopamine synthesis in the putamen and limbic striatum [44]. Large multicenter studies with standardized ICD phenotyping are required to further examine the plausible mechanisms underlying the interaction between ICDs and the *DRD3* risk polymorphism, as well as the role of other genetic and environmental factors.

#### 3.2.4. DRD4

The dopamine receptor D type 4 (*DRD4*) gene is located on chromosome 11p15.5 and encodes for DRD4 which is expressed in almost the same forebrain regions as the DR2 receptor and at lower levels in the cerebral cortex. *DRD4* contains a remarkable number of polymorphic regions. There is a hypervariable region in the third cytoplasmic loop with 2–11 imperfect 48 base pair repeats (48 bp VNTR) [29]. Individuals with seven or longer VNTRs (DRD4 7+) exhibit a higher risk for compulsive or addictive disorders, increased gambling or other similar behaviors [45,46]. It has been proposed that the *DRD4* 7+ VNTR forms heteromers with DRD2, enhancing dopamine-mediated inhibition of glutamate, affecting the tendency for ICDs [47]. Torres et al. highlighted the role of the DRD4 7+ polymorphism, as well as other demographic and clinical factors, including male gender, early disease onset, moderate and severe dyskinesia symptoms, sleep behavior disorders and psychiatric comorbidities, in the development of ICDs in PD patients [15]. In another study, a gene–drug interaction on gambling behavior was examined. More specifically, in a study performed in 200 healthy subjects, carriers of the 4/7 *DRD4* genotype had increased gambling propensity after levodopa administration (*p* < 0.01) [44], highlighting the importance of assessing genetic data when investigating the impact of different pharmacological agents on patients’ behavior.

#### 3.2.5. DDC

*DDC* is located on chromosome 7 and contains 15 exons and 14 introns. *DDC* encodes for the aromatic L-amino acid decarboxylase enzyme which is essential for dopamine synthesis via catalyzing the conversion of l-dihydroxyphenylalanine to dopamine. Moreover, DDC is involved in the synthesis of norepinephrine and serotonin via conversion of l–5 hydroxytryptophan to serotonin and l-tryptophan to tryptamine, respectively. Different mRNA transcripts, neuronal and non-neuronal, encoding the same proteins, are produced due to alternative splicing in the 5′ UTR [48]. In a recent study of 353 PD patients, the presence of allele C in *DDC* rs1451375 intron polymorphism was found to affect ICD susceptibility (z = 3.22, *p* = 0.001) [16]. Another variant in the promoter region, the heterozygous and homozygous genotype of rs3837091 (*p* = 0.01 and *p* = 0.04), and genotype AA was also found to be associated with ICDs in 276 PD patients who were under dopamine treatment [17]. Moreover, in the study by Erga et al., the *DDC*-rs4490786 intron polymorphism was also observed to increase the risk of ICDs [11], reinforcing the notion that polymorphisms in *DDC* probably alter the bioavailability of dopamine in the central nervous system (CNS) and regulate dopamine neurotransmission, especially in individuals under dopamine treatment [16].

#### 3.2.6. SLC22A1

The solute-like carrier family 22 member 1 (*SLC22A1*) gene, also known as *OCT1*, is located on chromosome 6q25.3; it consists of 11 exons that span 37 kb [18]. *SLC22A1* encodes the organic cation transporter 1 (OCT1) protein, which transports endogenous compounds, including dopamine, contributing to the distribution and regulation of dopamine levels [49]. Redenšek et al. studied 231 PD patients and observed that the carriers of the *SLC22A1* rs628031 AA genotype had higher odds for ICDs, suggesting a new predictive biomarker of ICDs in PD patients receiving dopaminergic treatment (OR = 3.16; 95% CI = 1.03–9.72; *p* = 0.045) [18]. Replication of these results in independent PD cohorts is needed in order to clarify the role of this genetic biomarker in the development of ICDs in PD patients treated with dopaminergic agents, facilitating a more individualized approach in PD management.

#### 3.2.7. DBH

The dopamine beta-hydroxylase (*DBH*) gene is located on chromosome 9q34.2. It encodes for a copper-containing enzyme that converts dopamine to norepinephrine.

In a recent study evaluating genetic markers as risk factors for ICDs in PD patients under dopaminergic therapy, the *DBH* rs1611115 polymorphism and particularly the recessive allele C were associated with ICD development (*p* = 1.1 × 10^−4^; OR = 3.51, 95% CI [1.77–7.29]). This substitution has been found to affect enzyme plasma activity [13]. Additional studies for the role of this or other *DBH* variants are needed to improve our understanding regarding the role of this gene in PD-ICDs.

### 3.3. Genes Involved in the Glutamatergic Pathway

The glutamatergic pathway is the primary excitatory neurotransmitter system in the CNS. It plays a critical role in the underlying pathophysiology of PD and ICDs, affecting how the brain handles reward, excitotoxicity and motor regulation. Glutamate, responsible for the majority of excitatory signals in the brain, binds to ionotropic (N-methyl-D-aspartate, NMDA) and amino-3-hydroxy-5-methyl-4-isoxazolepropionic acid (AMPA) or metabotropic glutamate (mGluRs) receptors [50].

#### GRIN2B

The glutamate receptor, ionotropic, N-methyl-d-aspartate 2B (*GRIN2B*) gene is located on chromosome 12p13.1, and it encodes for the NR2B subunit of the NMDA receptor. NMDA receptors are ionotropic glutamate receptors that participate in glutamate-mediated neurotransmission in the brain [10]. The NMDA receptor has been associated with PD, as alterations in the expression of NMDA receptor subunits or factors affecting NMDA receptor activation influence glutamate release, leading to the death of dopaminergic neurons. The NMDA receptor is composed of two subunits, NR1 (GRIN1) and NR2 (GRIN2). Regarding the NR2 subunit, it has four subtypes: GRIN2A, GRIN2B, GRIN2C and GRIN2D [8]. The GRIN2B subunit of the NMDA receptor is highly concentrated in the striatum/basal ganglia.

The *GRIN2B* rs7301328 (C366G) polymorphism has been associated with an increased risk of developing ICDs in PD patients (OR: 2.57, *p* = 0.0087) [8,17]. Notably, rs7301328 is a synonymous single nucleotide substitution; thus, although it alters the DNA sequence, it does not cause an amino acid change. Gene–gene interactions, especially between genes involved in the dopaminergic and glutamatergic pathways, would be of particular interest, as would the identification of miRNA-binding site domains.

### 3.4. Genes Involved in the Serotonergic Pathway

ICBs involve a kind of behavioral addiction, and mesolimbic dopaminergic and serotonergic pathways are believed to be closely connected and implicated in these addictive behaviors. Serotonin is a main neurotransmitter in the CNS, but also an important signaling molecule in the periphery. Serotonergic neurotransmission is widely distributed in the brain and is mediated by serotonergic neurons in the raphe nuclei, which project to the striatum. Serotonin homeostasis is mainly regulated by its receptors, transporter and enzymes of its biosynthetic pathway, including the 5-hydroxytryptamine receptor 2A (HTR2A) and tryptophan hydroxylase 2 (TPH2) [51].

#### 3.4.1. HTR2A

The 5-Hydroxytryptamine receptor 2A (*HTR2A*) gene is located on chromosome 13q14–q21 and encodes the 5-HT2A serotonin receptor, which is a G protein-coupled receptor (GPCR) that regulates neurotransmitter release, including glutamate and dopamine release, neuropsychiatric processes and peripheral effects [52]. In a case–control study, the *HTR2A* c.102T>C (rs6313) polymorphism was examined in 404 Korean PD patients for the risk of ICDs. The T allele was marginally associated with impulse control and repetitive behaviors in PD, and this effect was more profound in the lower levodopa-equivalent-dose PD patient group (*p* = 0.011 compared to *p* = 0.961 in the PD group receiving the higher levodopa-equivalent-dose) [20]. This variant has previously been associated with increased serotonin 2A receptor expression [53], increasing serotonin 2A receptor activity. In the study by Lee et al., the significant effect of the c.102T>C variant of *HTR2A* with ICD, in a dose-dependent manner, was also observed, suggesting a dose-dependent genetic susceptibility [20]. Additional studies with an increased number of patients and *HTR2A* polymorphisms are awaited to increase our understanding regarding the involvement of this gene in PD ICDs.

#### 3.4.2. TPH2

The tryptophan hydroxylase 2 (*TPH2*) gene is located on chromosome 12q21.1. It consists of 11 exons, and it encodes a rate-limiting enzyme in serotonin synthesis, primarily expressed in the brain. In the study by Redenšek et al., *TPH2* rs4290270 and TPH2 rs4570625 polymorphisms were studied. Carriers of the TPH2 rs4570625 GT genotype and carriers of at least one T allele were associated with ICDs in PD patients [18]. This allele has been shown to decrease serotonin synthesis [54]. Reduced serotonin levels may, in turn, reduce tonic inhibition of midbrain dopaminergic neurons, induce dopamine release, leading to ICDs [55]. Interestingly, specific *TPH2* haplotypes have also been associated with increased risk of ICDs, as well as a higher tendency toward risk-taking behavior, particularly in males [18]. Larger studies in different ethnicities are required to better understand the role of *TPH* in ICDs in PD.

### 3.5. Genes Involved in the Opioid Pathway

The opioid system can modulate dopaminergic pathways; opioids inhibit GABA production, thus releasing inhibition of dopamine release, leading to increased dopamine levels [11]. Opioid receptors include the mu1 receptor (OPRM1) and kappa1 receptor (OPRK1) which belong to the 7-transmembrane GPCR family that regulate pain, mood and physiological responses by binding to endogenous ligands like endorphins and exogenous drugs like fentanyl [56].

#### OPRK1 and OPRM1

OPRK1, which is located on chromosome 8q11.23, encodes the kappa-opioid receptor (KOR) and plays an important role in regulating the effects of endogenous opioids, as well as addiction-related behaviors, pain and stress. In addition, *OPRM1*, located on chromosome 6q24–q25, encodes the Mu-type opioid receptor that mediates the analgesic effects, reward and addictive properties of both endogenous and exogenous opioids. Opioids are fundamental factors in the addictive process, especially via the reward system and the reinforcement process. MORs are mainly associated with positive reinforcement, whereas KORs are associated with negative reinforcement [57].

In a clinical–genetic study of 276 PD patients aiming to predict the incidence of ICDs in early-stage PD, a panel of genetic variants, including *OPRM1* rs677830 and OPRK1 rs702764, improved the prediction of ICDs, reinforcing the clinical utility of genetic testing. In fact, *OPRM1* rs677830 and *OPRK1* rs702764, together with GRIN2B rs1105581, rs7301328, Catechol-O-methyltransferase (*COMT*) rs4646318, *TPH2* rs4290270, and Dopamine receptor D type 5 (*DRD5*) rs6283 polymorphisms were associated with a decreased risk of ICDs [17]. Thus, genetic panels of different genes can aid in the early identification of PD patients with increased risk of displaying ICDs. In another study, the results of the clinical–genetic prediction model were more robust in patients initiating DA therapy. More specifically, variations in *OPRK1*, *HTR2A* and *DDC* had the strongest genetic predictive effect [11]. Moreover, Verholleman et al. examined the possible impact of *OPRM1* rs1799971 polymorphism on the effectiveness of naltrexone on hypersexuality symptoms [21]. Naltrexone, a mu–delta–kappa antagonist, binds to the mu-opioid receptor (MOR), encoded by *OPRM1*. Interestingly, the A118G (rs1799971) *OPRMR1* polymorphism in exon 1 causes an amino acid exchange at residue 40 of the MOR; the normal asparagine (Asn, A allele) is changed to an abnormal aspartic acid (Asp) residue (G allele) (Asn40Asp) which decreases its expression and binding ability in the brain [58]. The relationship between clinicogenetic and pharmacologic risk factors for ICD in PD needs to be further investigated, as well as the functional significance of specific genetic risk factors.

### 3.6. Genes Associated with Monogenic PD

A number of genes that have been associated with familial PD have been recognized as candidate genes implicated in ICD pathology, suggesting possible common pathogenic mechanisms. However, the exact pathways have not been identified.

#### 3.6.1. LRRK2

The leucine-rich repeat kinase 2 (*LRRK2*) gene is located on chromosome 12q12. It encodes the dardarin protein which is involved in crucial cellular processes, such as vesicular trafficking, autophagy and cytoskeleton maintenance. *LRRK2* mutations have been recognized in familial, as well as in sporadic PD. The most prevalent *LRRK2* mutation is G2019S (rs34637584) [59].

Sun et al. studied 883 PD patients collected from the PPMA database and observed that PD patients with the *LRRK2* G2019S mutation had a higher impulse control disorder score compared to PD patients without the mutation (*p* = 0.027). However, although these PD patients were characterized by a more severe clinical presentation at the baseline, they had a slower rate of disease progression and a reduction in impulse control problems. Probably, these could be due to complex compensatory mechanisms and cellular restoration processes, environmental or other unknown yet factors that influence disease progression in *LRRK2* G2019S carriers [22]. Additional studies examining G2019S and other *LRRK2* variants are awaited to add to the total pool of data investigating the role of *LRRK2* in ICDs.

#### 3.6.2. FBXO7

The F-box protein 7 (*FBXO7*) gene is located on chromosome 22q12-q13. It encodes a protein involved in ubiquitin-mediated degradation and is a rare monogenic cause of autosomal recessive PD with a variety of clinical symptoms. Variations in this gene were first detected in an Iranian family by genome-wide linkage analysis. Since then, only seven types of pathogenic variants have been described. PD patients with *FBXO7* mutations have been found to have early-onset disease, ranging from 10 to 52 years, and akinetic-rigidity dominant parkinsonism with variable response to levodopa [60].

Recently, Yoo et al. described a PD patient with early-onset disease harboring two novel pathogenic *FBXO7* variants, a nonsense (c.1162C>T, p.Gln388X) and a missense (c.80G>A, p.Arg27His) one. During treatment with levodopa and a small dose of dopamine agonist, serious ICDs arose, limiting medical treatment [24]. Close monitoring for early manifestations of ICDs may be required in PD patients with FBXO7 mutations.

### 3.7. Other Genes

A limited number of genes that have been previously recognized as risk factors of PD have been found to be associated with ICDs; however, the implicated pathways remain to be elucidated.

#### 3.7.1. GBA

The glucosylceramidase beta (GBA) gene is located on chromosome 1q21. It encodes for the lysosomal enzyme glucocerebrosidase (GCase), which catalyzes the hydrolysis of glucocerebroside into glucose and ceramide. *GBA* mutations are the most common genetic factor increasing susceptibility to PD. *GBA* mutation carriers have been associated with an earlier PD onset, rapid disease progression and an increased burden of non-motor symptoms, such as cognitive dysfunction, rapid eye movement sleep behavior disorder and hyposmia [61].

In the study by Amami et al., which included 46 PD patients, ICDs had a higher incidence in GBA-PD patients (52.2%) compared to non-mutated PD patients (13%), with hypersexuality and compulsive shopping being the most prevalent ones. Only one PD patient was on levodopa monotherapy, and most of them were taking dopamine agonists [25]; other studies have also shown increased prevalence of ICDs in GBA-PD patients compared to sporadic PD, whereas some showed no difference [62,63,64,65].

#### 3.7.2. APOE

The apolipoprotein E (*ApoE*) gene is located on chromosome 19q13.32, and it encodes a multifunctional protein involved in lipid transport, cholesterol metabolism and nerve repair. ApoE has three major isoforms: ApoE2, ApoE3, and ApoE4, which are expressed by the polymorphic alleles ε2, ε3, and ε4. The amino acid change within the ApoE isoforms affects protein stability and interactions, with the ApoE4 isoform being the least stable [66].

The *APOE* ε4 allele is the strongest genetic risk factor for Alzheimer’s disease and is also associated with α-synuclein pathology and faster cognitive decline in PD patients [67,68]. Recently, Chen et al. studied 87 PD patients from the Parkinson’s Progression Markers Initiative over a time period of 5 years and found a strong connection between the *APOE* ε4 allele and accelerated ICD progression in newly diagnosed PD patients (HR  =  1.450, *p*  =  0.048), suggesting a plausible relationship between the *APOE* ε4 allele and progression of ICDs in PD [26].

#### 3.7.3. BDNF

The brain-derived neurotrophic factor (*BDNF*) gene is located on chromosome 11p13–14. BDNF is essential for neuron survival, growth, and synaptic plasticity. *BDNF* encodes for a large promolecule (pro-BDNF) with a secretory signal peptide. The Val66Met (rs6265) polymorphism in the 50-pro-BDNF sequence changes the intracellular tracking and packaging of pro-BDNF, affecting the function and production of the mature BDNF protein [69]. The dominant Allele A of the rs6265 substitution has recently been found to increase the risk of ICD development in 386 PD patients (*p* = 5.7 × 10^−6^, OR = 4.49, 95% CI [2.24–9.18]) [13]. Larger studies in diverse populations are needed to clarify the role of BDNF in PD-related ICDs.

#### 3.7.4. ACE

Angiotensin I-converting enzyme (*ACE*) gene is located on chromosome 17q23.3 and encodes the angiotensin-converting enzyme peptidyl dipeptidase A. ACE has a fundamental role in the Renin–Angiotensin–Aldosterone System (RAAS) which regulates blood pressure and fluid and electrolyte balance in the human body [70]. In fact, ACE acts as a dipeptidyl carboxypeptidase that converts the inactive decapeptide angiotensin I into the active octapeptide angiotensin II.

The common insertion/deletion (rs4646994) polymorphism in intron 16 of *ACE* is seen as an insertion (I) and/or deletion (D) of a sequence of Alu repeats with a length of 289 bp. In a study based on the genotypes of 45 PD patients [13], the dominant allele I has recently been associated with ICD in PD (OR 55.17, CI95% 6.80–447.57).

#### 3.7.5. NOS1

Nitric Oxide Synthase 1 (*NOS1*) gene is located on chromosome 12q24.2-q24.31 and encodes an enzyme that produces nitric oxide, which acts as a neurotransmitter in the brain and peripheral nervous system and is implicated in neurotoxicity as well. Recently, the *NOS1* rs2682826 A allele was found to increase the susceptibility to ICDs [18,27].

### 3.8. Research Gaps and Future Prospects

**Figure 2:**
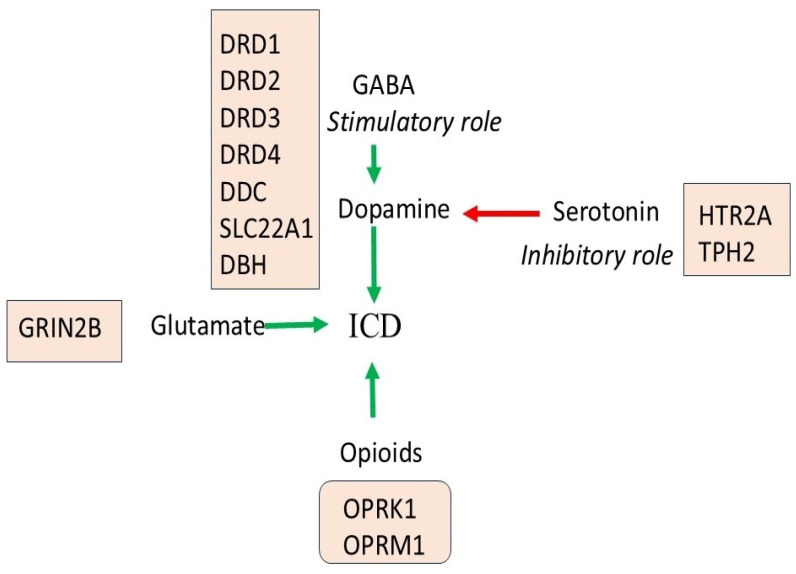
Schematic diagram of the pathways and genes that contribute to ICDs in PD. Green and red arrows indicate stimulatory and inhibitory roles respectively.

ICD is a complex process, the pathophysiology of which remains currently elusive. A schematic diagram that depicts major pathways, genes, and their involvement in ICDs is presented in Figure 2. Dopaminergic treatment, especially with dopaminergic agonists, has been associated with the development of ICDs; however, not all PD patients suffer from these behavioral addictions. The reported prevalence of ICDs varies, as there are no standard screening tools, many cases are often undiagnosed, and no long-term universal management guidelines are followed. The socioeconomic burden of ICDs is also understudied. Moreover, there is usually a lack of a multidisciplinary approach which is essential, as ICDs are characterized by many psychiatric comorbidities. ICDs are often treated as a single entity; however, multiple ICD subtypes exist, and the role of genetic and other biomarkers seems to be crucial in this discrimination. Thus, there is a need to identify specific genetic, epigenetic or protein biomarkers for certain ICD subtypes in order to follow a more personalized PD treatment approach. The exact pathological mechanisms leading to ICDs remain currently elusive; however, the combined effects of individual polymorphisms in genes that participate in different pathways could provide useful information regarding ICD pathology. Future gene–gene and gene–environmental studies, as well as functional studies, are awaited to further improve our understanding of the pathophysiology of ICDs in PD. Moreover, larger studies in different ethnicities need to be carried out in order to draw more definite conclusions. Exome sequencing information is required to clarify which genes are involved and which pathways are implicated, followed by well-designed replication studies. Functional neuroimaging data will also enhance our understanding of ICDs’ pathology. Thus, the integration of genetic data with neuroimaging and clinical phenotypes may improve predictive models and hopefully facilitate personalized treatment of PD patients.

Among the currently identified genetic factors, dopaminergic pathway genes seem to have the strongest association with ICD susceptibility in PD, in particular, variants involving dopamine receptors and dopamine metabolism. On the other hand, studies on the genes implicated in the glutamatergic, serotonergic, and opioid pathways are limited, and evidence is less consistent. Dopaminergic treatment may further increase the effects of pre-existing genetic susceptibility within the above-mentioned interconnected pathways, contributing to ICD development in PD patients. Currently there is no single genetic marker that demonstrates sufficient predictive value in order to be used in clinical practice; however, combinations of genetic variants together with clinical and pharmacological factors may improve risk stratification strategies.

### 3.9. Integrative Pathophysiological Model of ICDs in PD

The development of ICDs in PD is unlikely to be explained by single genetic variants. Instead, current evidence supports a model in which multiple neurotransmitter systems interact to influence reward processing, behavioral reinforcement, and impulse control. The relationship between dopaminergic, glutamatergic, serotonergic, and opioid systems is a complex, interconnected network within the brain’s mesolimbic reward pathway (ventral tegmental area) to the nucleus accumbens. Dopaminergic dysfunction, which often arises from the overstimulation of dopamine receptors, especially D3 receptors located in the mesolimbic system, is a primary driver in the development of ICDs. This is particularly evident when people are taking dopamine agonist therapy; however, other neurotransmitters may be involved in this imbalance, such as serotonin and glutamate [71].

The glutamatergic system contributes to synaptic plasticity and reward-based learning, with gene variants such as GRIN2B potentially influencing excitatory signaling and behavioral adaptation. Serotonin and dopamine share strong anatomical and functional interactions. Serotonergic pathways such as HTR2A and TPH2 regulate inhibitory control and impulsivity, and reduced serotonergic tone may disinhibit dopaminergic activity [72], promoting impulsive behavior.

Moreover, the opioid system modulates reward sensitivity and reinforcement by influencing dopamine release via GABAergic mechanisms. Variations in the *OPRM1* and *OPRK1* genes are strongly linked to altered susceptibility to addictive and compulsive behaviors.

As a result, it seems that ICDs in PD may be attributed to a multi-system imbalance, where genetic susceptibility affects the interaction between dopaminergic, glutamatergic, serotonergic, and opioid pathways, resulting in dysregulation of reward processing and impulse control.

Moreover, the association between gene polymorphisms in dopamine and opioid receptors and ICDs is involved in what is known as “reward deficiency syndrome,” a condition where people experience reduced feelings of satisfaction due to the interaction between the genes and environment [11].

This system-level perspective may help explain inter-individual variability and highlight the need for integrative, rather than gene-centric, approaches in future research.

## 4. Conclusions

ICDs are an increasingly recognized non-motor complication in PD. Notably, ICDs are often seen in patients treated with dopaminergic agents, especially dopamine agonists; however, this is not the case for all PD patients. Specific clinical, genetic or environmental factors seem to have a pivotal role in ICD pathogenesis. The genetic jigsaw of ICDs is complicated; however, several genes that encode for enzymes, transporters and receptors that participate in dopamine synthesis, transport, degradation and signaling have been found to be associated with the development of ICDs in PD patients, underlying a fundamental role of the dopaminergic pathway in ICD pathology. *GRIN2B,* which is involved in the glutamatergic pathway, may also have a critical role in the underlying pathophysiology of PD-ICDs, affecting mainly the reward system. Serotonergic neurotransmission is believed to be closely related to these behavioral addictions as well; notably, variations in *HTR2A* and *TPH2* have already been found to increase susceptibility for PD-ICDs. Moreover, the opioid system that is known to interact with the dopaminergic system is another potential crucial factor in ICD pathogenesis, with *OPRK1* and *OPRM1* being recognized as susceptibility genes.

To conclude, ICDs are complex neurobehavioral complications of PD influenced by multiple factors. Genetic variability across multiple neurotransmitter systems appears to contribute to individual susceptibility, although current evidence remains contradictory. Future research should include large, multicenter studies with well-defined cohorts and standardized ICD phenotyping in order to improve comparability and adopt integrative approaches that combine genetic, clinical, and neurobiological data to improve risk prediction and enable personalized treatment strategies.

## Figures and Tables

**Table 1:** Genes and pathways implicated in PD-ICDs.

Gene	Chromosome	Variant	Main Pathway Affected	References
***DRD1***	5q35.1	rs4532, rs4867798, rs5326	Dopaminergic pathway	[9,10,11]
***DRD2***	11q22-23	rs1800497, rs6275, rs12364283, rs1076560	Dopaminergic pathway	[10,12,13]
***DRD3***	3q13.3	rs6280	Dopaminergic pathway	[14]
***DRD4***	11p15.5	VNTRs (DRD4 7+)	Dopaminergic pathway	[15]
***DDC***	7p12.2-p12.1	rs1451375 rs3837091 rs4490786	Dopaminergic pathway	[11,16,17]
***SLC22A1***	6q25.3	rs628031	Dopaminergic pathway	[18]
***DBH***	9q34.2	rs1611115	Dopaminergic pathway	[13]
***GRIN2B***	12p13.1	rs7301328	Glutaminergic pathway	[10,19]
***HTR2A***	13q14–q21	rs6313	Serotonergic pathway	[20]
***TPH2***	12q21.1	rs4290270, rs4570625	Serotonergic pathway	[18]
***OPRK1***	8q11.23	rs702764	Opioid pathway	[11,17]
***OPRM1***	6q24–q25	rs677830, rs1799971	Opioid pathway	[17,21]
***LRRK2***	12q12	rs34637584	unknown	[22]
***SNCA***	4q21.3-q22	unknown	unknown	[23]
***FBXO7***	22q12-q13	c.1162C>T, c.80G>A	unknown	[24]
***GBA***	1q21	unknown	unknown	[25]
***APOE***	19q13.32	*APOE* ε4 allele	unknown	[26]
***BDNF***	11p13–14	rs6265	unknown	[13]
***ACE***	17q23.3	rs4646994	unknown	[13]
***NOS1***	12q24.2-q24.31	rs2682826	unknown	[18,27]

## Data Availability

No new data were created or analyzed in this study. Data sharing is not applicable to this article.

## Abbreviations

The following abbreviations are used in this manuscript:

ICDs	Impulse control disorders
PD	Parkinson’s disease
LBs	Lewy bodies
DDC	Dopa decarboxylase
5′-UTR	5′ untranslated region
ANKK1	Ankyrin repeat and kinase domain containing 1
DRD3	Dopamine receptor D type 3
DRD4	Dopamine receptor D type 4
SLC22A1	Solute-like carrier family 22 member 1
CNS	Central nervous system
DBH	Dopamine beta-hydroxylase
NMDA	N-methyl-D-aspartate
AMPA	Amino-3-hydroxy-5-methyl-4-isoxazolepropionic
mGluRs	Metabotropic glutamate receptors
GRIN2B	Glutamate receptor, ionotropic, N-methyl-d-aspartate 2B
HTR2A	Hydroxytryptamine receptor 2A
TPH2	Tryptophan hydroxylase 2
GPCR	G protein-coupled receptor
OPRK1	Opioid receptor kappa 1
OPRM1	Opioid receptor Mu 1
COMT	Catechol-O-methyltransferase
DRD5	Dopamine receptor D type 5
LRRK2	Leucine-rich repeat kinase 2
SNCA	a-synuclein
FBXO7	F-box protein 7
GBA	Glucosylceramidase beta
APOE	Apolipoprotein E
BDNF	Brain-derived neurotrophic factor
NOS1	Nitric Oxide Synthase 1
